# Tissue Specific Diurnal Rhythms of Metabolites and Their Regulation during Herbivore Attack in a Native Tobacco, *Nicotiana attenuata*

**DOI:** 10.1371/journal.pone.0026214

**Published:** 2011-10-18

**Authors:** Sang-Gyu Kim, Felipe Yon, Emmanuel Gaquerel, Jyotasana Gulati, Ian T. Baldwin

**Affiliations:** Department of Molecular Ecology, Max Planck Institute for Chemical Ecology, Jena, Germany; University of California, United States of America

## Abstract

Ecological performance is all about timing and the endogenous clock that allows the entrainment of rhythms and anticipation of fitness-determining events is being rapidly characterized. How plants anticipate daily abiotic stresses, such as cold in early mornings and drought at noon, as well as biotic stresses, such as the timing of pathogen infections, is being explored, but little is known about the clock's role in regulating responses to insect herbivores and mutualists, whose behaviors are known to be strongly diurnally regulated and whose attack is known to reconfigure plant metabolomes. We developed a liquid chromatography-mass spectrometry procedure and analyzed its output with model-based peak picking algorithms to identify metabolites with diurnal accumulation patterns in sink/source leaves and roots in an unbiased manner. The response of metabolites with strong diurnal patterns to simulated attack from the specialist herbivore, *Manduca sexta* larvae was analyzed and annotated with in-house and public databases. Roots and leaves had largely different rhythms and only 10 ions of 182 oscillating ions in leaves and 179 oscillating ions in roots were rhythmic in both tissues: root metabolites mainly peaked at dusk or night, while leaf metabolites peaked during the day. Many oscillating metabolites showed tissue-specific regulation by simulated herbivory of which systemic responses in unattacked tissues were particularly pronounced. Diurnal and herbivory-elicited accumulation patterns of disaccharide, phenylalanine, tyrosine, lyciumoside I, coumaroyl tyramine, 12-oxophytodienoic acid and jasmonic acid and those of their related biosynthetic transcripts were examined in detail. We conclude that oscillating metabolites of *N. attenuata* accumulate in a highly tissue-specific manner and the patterns reveal pronounced diurnal rhythms in the generalized and specialized metabolism that mediates the plant's responses to herbivores and mutualists. We propose that diurnal regulation will prove to an important element in orchestrating a plant's responses to herbivore attack.

## Introduction

Timing is everything for ecological performances. The earth's 24 h rotation on its tilted axis, geographical differences, and interactions with other organisms shape the specific diurnal rhythms of each organism. As sessile organisms, plants can entrain their physiology to abiotic condition of their environment such as day/night cycles and their associated temperature fluctuations. The endogenous plant clock (circadian clock) ‘wakes up’ the photosynthetic machinery just before sun rise to maximize energy harvesting and regulates guard cells at noon to minimize water loss [1]. It also increases cold tolerance of plants at night and dawn [2]. Plants also synchronize their physiology with tightly associated organisms. For instance, snapdragon flowers emit methyl benzoate during the day to attract day-active pollinating bees [3]. Pathogens that attack at dawn are anticipated by the clock in Arabidopsis [4].

Since the pioneering discoveries that the rhythmic behaviors of animals are encoded in their genomes, the molecular components and functions of the plant's endogenous clock have been identified in the model plant, *Arabidopsis thaliana* [5]. Forward and reverse genetic approaches in Arabidopsis have revealed that many diurnal ‘behaviors’ are controlled by a few clock genes [1], [5]. These clock genes regulate 30∼40% of total gene expression in Arabidopsis [6]. Arrhythmic plants harboring mutations in the clock genes have reduced photosynthetic capacity, growth and competitive ability under normal conditions [7], [8]. However, natural mutations in clock genes have been discovered that help entrain a particular accessions' physiology to its local environment which enhances the plant fitness in that area [8].

Plants fix carbon in the shoot using light energy and make numerous metabolites from the products of photosynthesis. Plant metabolites therefore originate from a day/night cycle. Studies of primary metabolites have shown that sugars, starch, amino acids, most of organic acids involved in photosynthesis are circadian-regulated in leaves [9]. Although transcriptomic analyses show that many genes involved in secondary metabolite biosynthesis have diurnal expression patterns [5], the diurnal rhythms of secondary metabolite levels have been less studied in comparison with those of primary metabolites.

Plants are exposed to two completely different environments: the aboveground and the belowground. Aboveground and belowground plant parts thus develop their own endogenous rhythms [10]. Clock components in the shoot and root oscillate in a similar way under light/dark cycles but not under constant light. The roots express approximately four-fold fewer oscillating genes than do shoots in Arabidopsis [10]. However, this does not mean that the endogenous clock is less important for root physiology. Important physiological processes such as root bending and lateral root formation occur every 6 h and are controlled by the internal clock [11]. In addition, clock-regulated water contents in roots are reduced during the day and increase during the night [12].

*Nicotiana attenuata* is a native tobacco plant growing in the Great Basin Desert of southwestern USA. It germinates in post-fire ecosystems and shows diverse intra-specific and inter-specific interactions. We have studied *N. attenuata* growing in its ecological niche in natural habitats for more than two decades and observed several diurnal rhythms in its physiology. *N. attenuata* interacts with different pollinators in a light-dependant manner [13], [14]. It produces two kinds of flowers. Night-opening flowers (NoFs) that open their corollas during the night and close during the day. Morning-opening flowers (MoFs) open their corollas during the early morning. NoFs emit benzyl acetone only during the night to attract nocturnal hawkmoth pollinators (*Manduca sexta* and *M. quinquemaculata*). Early morning flower visitors such as humming birds mainly nectar at MoFs and transfer pollen. Plant-herbivore interactions are also regulated in a day/night cycle. The accumulation of two lipoxygenase trancripts (*NaLOX2* and *NaLOX3*) involved in the biosynthesis of green leaf volatiles and jasmonic acid (JA) show diurnal rhythms in the leaf [15]. In addition, the generalist predator, *Geocoris* spp. feeds on the eggs and neonates of the specialist herbivore, *M. sexta*, usually during the day.

Metabolites produced in leaves and roots are essential elements determining the outcome of plants' aboveground and belowground interaction with other organisms. If most of the organisms on earth are governed by their endogenous clocks, the rhythms of the metabolites they produce should help us understand plant-plant, plant-animal interactions. Even though many genes involved in metabolism show diurnal expressions, large-scale screenings of tissue-specific oscillating metabolites and their regulation by herbivory remain largely unknown. Here, we examined tissue-specific diurnal rhythms of metabolites in leaves and roots of *N. attenuata* for two days. To find interconnections among oscillating metabolites and herbivore-induced plant defenses, we treated mechanical wounds in the leaves of *N. attenuata* with oral secretions (OS) from *M. sexta* larvae to mimic herbivore-induced changes [16] and precisely time the onset of elicitations to analyze the changes in oscillating metabolite levels with the oscillations of the transcripts of their associated genes.

## Results and Discussion

### Experimental Design

**Figure 1:**
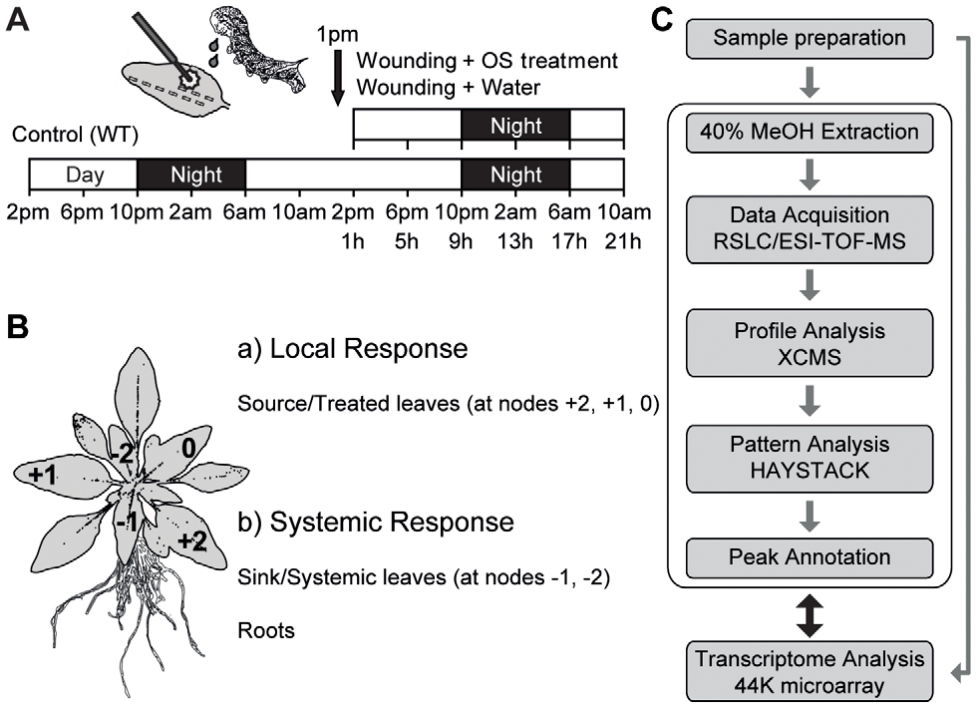
Experimental procedures used to identify oscillating and herbivore-induced metabolites and their associated genes in different tissues of *Nicotiana attenuata*. (A) Wild type (WT) *N. attenuata* plants were harvested every 4 h for two days during the initiation of stem elongation. To mimic herbivory, oral secretions (OS) of the larvae of the specialist herbivore, *M. sexta*, were immediately applied to puncture wounds made in leaves at 1 pm. Water treatment of puncture wounds on separate plants was used to distinguish OS-specific from wound-induced changes in metabolites and transcripts. (B) Metabolites from three different tissues, source leaves, sink leaves, and roots of *N. attenuata* were isolated. The leaf at node 0 had completed the sink to source transition and the leaf at node +1 was older by one leaf position than the leaf at node 0 and so forth. Source leaves (at nodes +2, +1, 0) were wounded with a fabric pattern wheel and treated with 20 µl of *M. sexta* OS, which was diluted 1∶5 with water. Untreated leaves (at nodes −1, −2) and roots were harvested to monitor systemic responses. (C) After sample preparation from six biological replicates, a 40% methanol extraction method optimized for the defense metabolites of *N. attenuata* was used and the metabolites separated with a rapid separation liquid chromatography (RSLC) on a C_18_ column and detected by ESI-TOF-MS (electrospray ionization time-of-flight mass spectrometer) for parents and their daughter ions. Peak picking and alignments were performed with the XCMS package [19]. Diurnal oscillating metabolites were extracted by the pattern matching algorithms of HAYSTACK tool [20]. In-house and public databases were used to identify oscillating metabolites and a 44K Agilent microarray designed for *N. attenuata* was used to examine the expression of metabolite-related genes.

*N. attenuata* plants were grown in 16 h light/8 h dark cycle for 5 weeks, and source/sink leaves and roots were collected every 4 h for two days without any treatment to identify oscillating metabolites (Figure 1A). Diluted OS (1∶5 with distilled water) from larvae of the specialist herbivore, *M. sexta*, were applied to puncture wounds in leaves created with a pattern wheel (W+OS) to mimic herbivory at 1 pm on a second day (Figure 1A). We also treated wounds with water (W+W) to distinguish OS-specific responses from wound-induced responses. Two source leaves (at nodes +2, +1) and one transition leaf (at node 0) were treated with water or OS, and collected to examine local response in oscillating metabolites and transcripts. We also collected two sink leaves (at nodes −1, −2) and roots to examine systemic response in untreated tissues (Figure 1B).

The enormous diversity of metabolites among different plant taxa and their diverse chemical properties means that metabolomic analysis must be optimized for each plant species. In previous studies, we developed an efficient method to extract defense-related metabolites in *N. attenuata* [17], [18] and used this 40% methanol-based extraction method (Figure 1C) and separated the metabolites by rapid separation liquid chromatography (RSLC). The separated metabolites were ionized by electrospray in both negative and positive modes and the exact mass to charge ratio of ions was measured with time of flight (TOF)-mass spectrometry. We also used a cDNA library and a microarray system of *N. attenuata* to explore the overall molecular mechanism in plant-herbivore interactions. The three-dimensional data (retention time, mass, intensity) of the mass spectrometry analysis was processed using the peak picking freeware XCMS [19], and diurnally oscillating metabolites were extracted by a model-based peak picking algorithm of the HAYSTACK [20] program. To reduce the information redundancy in the dataset, isotope peaks were clustered and annotated using the pseudo-spectrum deconvolution freeware CAMERA and removed from the analysis. Fragment and adduct ions detected in negative mode were included as there is no certain way to date of selecting only mother ions in large scale experiments, and negative ionization produces fewer daughter ions and adducts compared to positive ionization mode. In the negative ionization analysis, a total of 2209 and 1463 ions were detected from leaves and roots, respectively. With these platforms, we identified diurnally oscillating metabolites and their related transcripts accumulation (Figure 1C).

### Leaves and roots have distinctive diurnal patterns

**Figure 2:**
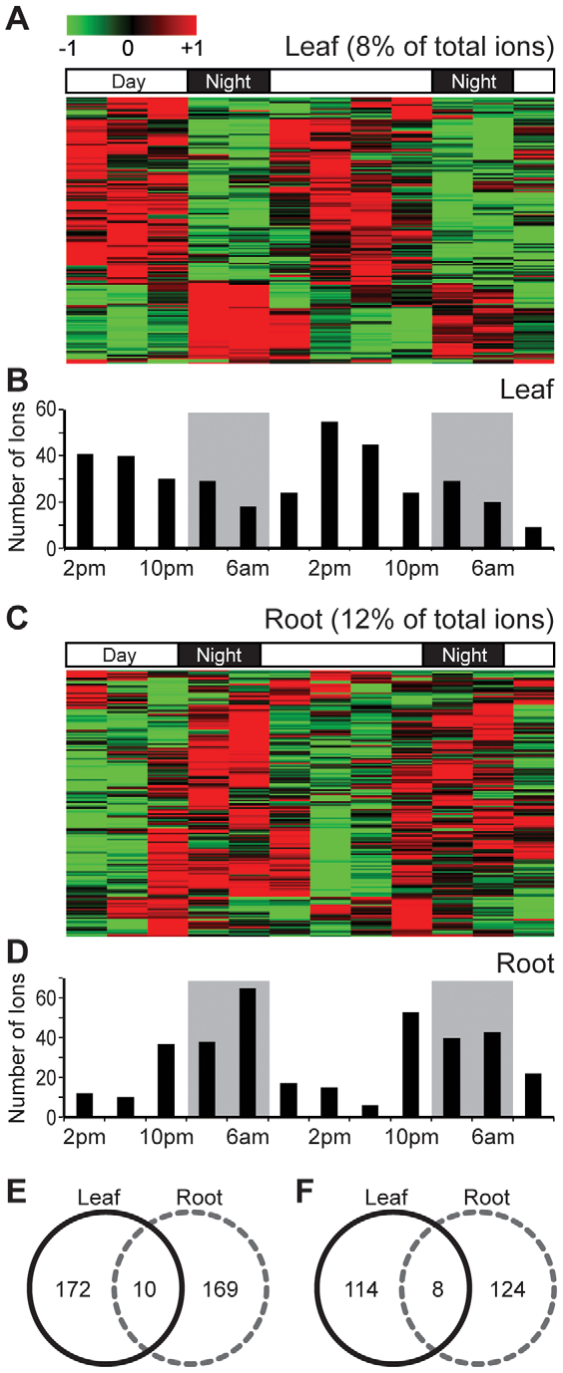
Accumulation of oscillating metabolites in *N. attenuata* show tissue-specific rhythms. Oscillating metabolites in source leaves (A) and roots (C) were roughly divided into two groups, one peaking during the day, and the other peaking at night. The heat map displays all of the Z-transformed oscillating metabolites levels in a false-color scale where green indicates low and red indicates high values. Each metabolite is represented by a single row with the average linkage hierarchical clustering tree obtained using Euclidean distances as metric. We counted the number of ions (y-axis) that peak at a particular harvest times (x-axis) in source leaves (B) and roots (D). Gray boxes depict the dark period. (E) Venn diagram of the oscillating metabolites selected across source leaves (black solid line) and roots (gray dashed line). (F) Venn diagram of the oscillating metabolites after removing adduct and daughter ions. CAMERA package and Pearson correlation [17] were used to select adduct and daughter ions detected from extracts of source leaves (black solid line) and roots (gray dashed line).

Pattern analysis revealed that 8% of total leaf metabolites and 12% of total root metabolites detected in negative mode had diurnal rhythms (Figure 2, Table S1 and File S1). Oscillating metabolites separated roughly into two groups by hierarchical clustering (Figure 2A and 2C). One group of metabolites was highly induced during the day, and the other group peaked at dusk or night. In leaf extracts, 72% of oscillating metabolites peaked during the day (Figure 2A), whereas 81% of root oscillating metabolites peaked at dusk or night (Figure 2C). The number of ions that show highest accumulations at each time clearly showed distinctive patterns in the two different tissues (Figure 2B and 2D). Moreover, only 10 ions (among 182 in leaves and 179 in roots) had diurnal accumulations in both tissues (Figure 2E) and among them, only one ion had the same diurnal rhythm. After the pattern analysis, we identified the fragment and adduct ions among the oscillating ions by CAMERA and by Pearson correlations dependent upon time and treatments [17] and found that a still smaller number of ions (8 ions among 122 and 132 ions in leaves and roots, respectively) were diurnal-regulated in both tissues (Figure 2F).

The results show that oscillating metabolites in *N. attenuata* accumulated in a tissue-specific manner and a few metabolites were commonly oscillating in both leaves and roots (Figure 2). According to the transcriptome analyses performed in root and shoot of Arabidopsis, the root has fewer oscillating genes than does the shoot under constant light condition [10]. However, our analysis of oscillating metabolites, which are the final products of gene regulations, demonstrates that roots also have strong diurnal rhythms which are distinct from those found in the leaves of *N. attenuata*.

Next, we annotated the oscillating ions using in-house and public databases, and analyzed their accumulation after W+W and W+OS treatments. Here, we describe the stories of 7 suites of oscillating metabolites, tales that speak of interesting interactions among the diurnal rhythms of metabolites and plant defense responses against herbivory.

### A disaccharide and its related genes

**Figure 3:**
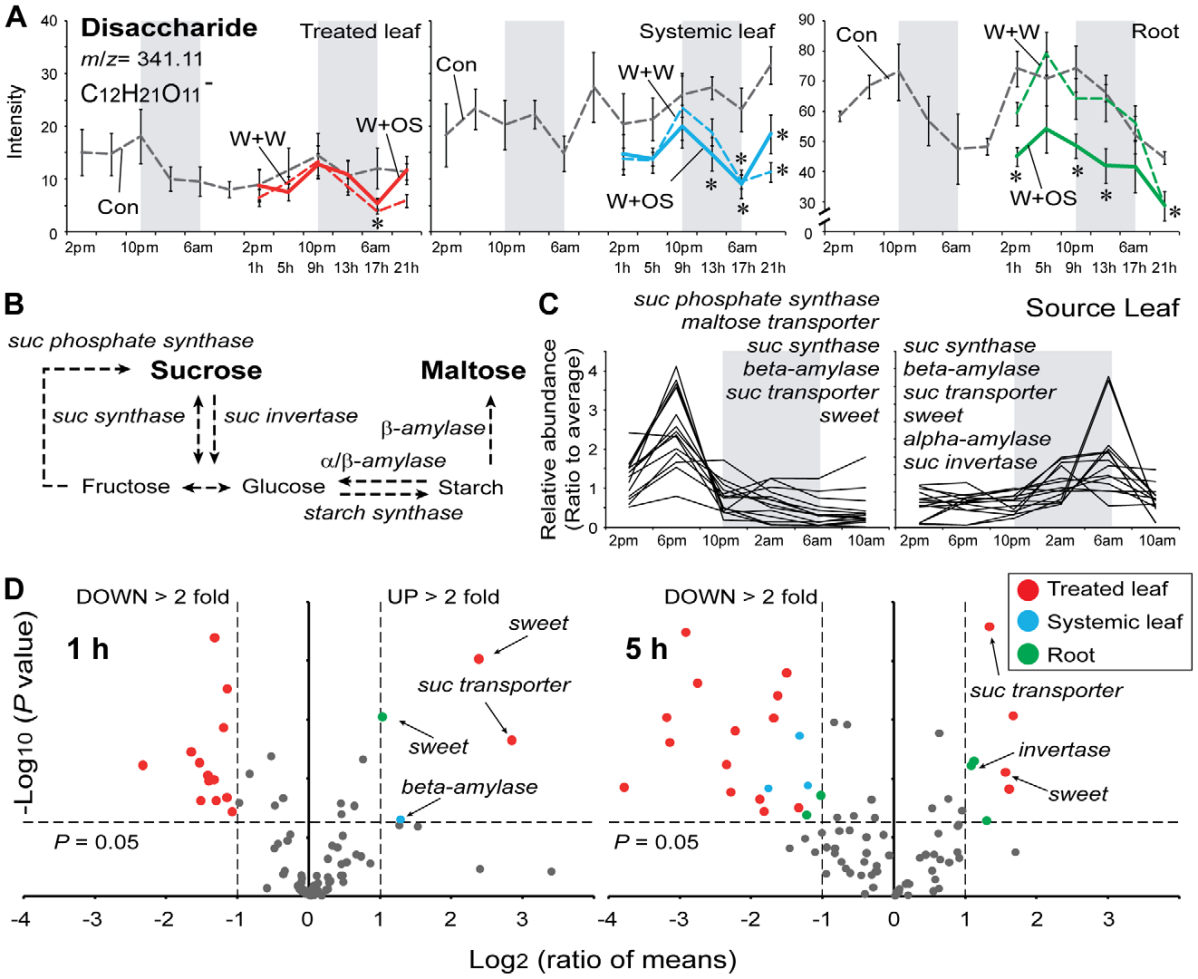
Accumulation of disaccharides and sugar related genes in three different tissues. (A) Mean (±SE) levels of normalized intensity of disaccharides (*m*/*z* 341.11 at 90 s, C_12_H_21_O_11_^−^) in source leaves, sink leaves and roots at each harvest time for two days (gray dashed lines) in control (Con) plants. After wounding and treating puncture wounds with either water (W+W, dashed lines with colors) or *M. sexta* OS (W+OS, solid lines with colors), disaccharides levels were examined in treated leaves (red), untreated systemic leaves (blue) and roots (green). Gray boxes depict the dark period. Asterisks indicate significant differences among the treatments at the indicated harvest time (* = *P*<0.05, one-way ANOVA with Bonferroni *post hoc* test). (B) Schematic overview of sucrose (suc) metabolism. (C) Two diurnal patterns of sugar metabolism-related genes (Table S2) accumulation in source leaves. Gray boxes depict the dark period. Sweet: sugar exporter [23]. Ratio to average: Ratio of transcript abundance at the time point shown, to the mean abundance of the same transcript across all time points. (D) Volcano plot analysis of the transcript levels of sugar-related genes in treated leaves (red dot), systemic untreated leaves (blue dot) and roots (green dot) 1 and 5 h after *M. sexta* OS-elicitation. The log_2_ ratio of mean intensities (OS-elicited/Con, with microarray expression data) plotted against the negative log_10_-transformed *P* value derived from Student's *t* tests. The horizontal dashed line indicates the threshold for statistically significant expression at *P* = 0.05 and the vertical dashed line, the threshold for two-fold changes in gene expression.

We first focused our attention on two signals corresponding to the *m*/*z* 341.11 and its dimer, *m*/*z* 683.23, at 90 s both with the same strong diurnal rhythms that peaked at dusk only in roots (Figure 3A and S1). While, the same signals were detected in treated and systemic leaves they did not pass our selection filter in these tissues (Figure 3A). We calculated the elemental formulas (*m*/*z* 341.11, C_12_H_21_O_11_^−^; *m*/*z* 683.23, C_24_H_43_O_22_^−^) using SmartFormula. Molecular mass and standard compound injections verified that *m*/*z* 341.11 at 90 s is a disaccharide and *m*/*z* 683.23 at 90 s is a dimer of the disaccharide.

Changes in primary metabolism strongly influence plant-herbivore interactions [21]. We therefore examined the regulation of disaccharide levels with their related genes (Figure 3B) after W+W and W+OS treatments. The result showed that only OS-elicitation rapidly reduced disaccharide levels in roots (Figure 3A, green line) within 1 h (*P*<0.05, one-way ANOVA followed by Bonferroni *post hoc* test). In untreated systemic leaves, disaccharide levels (Figure 3A, blue line) were significantly reduced by W+OS treatment after 13 h and W+W treatment after 17 h (*P*<0.05, one-way ANOVA followed by Bonferroni *post hoc* test). However, disaccharide accumulations in treated leaves did not change within 21 h after treatments (Figure 3A, red line), except for a small reduction at 17 h after W+W treatment (*P* = 0.0134, one-way ANOVA followed by Bonferroni *post hoc* test).

Most of the genes involved in sugar metabolism have diurnal rhythms [9], [22]. We blasted the Arabidopsis and other plant species homologues of these genes (Figure 3B) against our cDNA database of *N. attenuata* and identified the transcripts with high similarity (Table S2). We observed two distinct diurnal patterns of transcript accumulation for these genes using the HAYSTACK algorithm (Figure 3C and File S1). The first group peaked in the middle of the day and was low during the night (Figure 3C, left). The second group peaked at dawn and remained at low levels for the rest of the day (Figure 3C, right). We analyzed the regulation of these genes after OS-elicitation. Interestingly, the genes involved in sucrose transport (*suc transporter* and sugar exporter, *SWEET* [23]) were significantly up-regulated and several other genes (*β-amylase*, *suc synthase*, *suc phosphatase* and sugar exporter) were down-regulated by W+OS treatment in treated leaves (Figure 3D; *P*<0.05, Student's *t*-test), while disaccharide levels in treated leaves (Figure 3A) were not changed. The first group of genes peaked in the day was usually down-regulated in treated leaves by OS-elicitation but the regulation of the second group by OS-elicitation was highly variable (File S1). This pattern of regulation might be affected by the timing of induction (1 pm in this experiment). It will be interesting to evaluate in future studies how elicitations at night influence these patterns. In systemic leaves and roots, these genes were less regulated (Figure 3D), whereas suc levels reduced quickly after treatments (Figure 3A). However, root *SWEET* and *suc invertase* transcript accumulations were rapidly increased within 5 h of OS-treatment (Figure 3D; *P*<0.05, Student's *t*-test). The increase in *suc invertase* transcript levels is consistent with previous results of increased invertase activity in roots after OS-treatment [24].

Sucrose is synthesized from photosynthesis during the day and loaded into the phloem for transport to sink leaves and roots [25]. Starch is synthesized from sugars during the day and degraded into maltose to provide energy for the metabolic requirements of the dark phase [26]. A plant's clock anticipates the end of the night and regulates the rate of starch degradation to prevent energy limitations at night [27]. The sugar content in plants is known to have a diurnal rhythm [28]. A weak diurnal rhythm of disaccharide in source leaves was observed in early-elongated stage of *N. attenuata* but did not pass our selection filter (Figure 3A). The fact that our analytical procedures do not distinguish sucrose from maltose, which has a rhythm opposite to that of sucrose, may be the explanation, because sucrose accumulations are known to peak at dusk, while maltose peaks at night [27]. Testing this possibility will require calculating the ratio of sucrose and maltose in *N. attenuata* and examining separately their regulation during herbivory. The other reason may be the relatively strong selection filter that we employed. Consistent with this, the kinetic of this disaccharide in source leaves but not in sink leaves was detected as diurnally rhythmic when the stringency was lowered (data not shown).

OS-elicitation significantly altered many sugar-metabolic and transporter genes in treated leaves within 5 h (Figure 3D) but the disaccharide levels in treated leaves was not changed significantly within 21 h (Figure 3A). This indicates that the rapid turnover of disaccharide levels may maintain sugar levels in treated leaves, which is used to produce secondary metabolites for direct defense responses and supply carbon for allocation from shoot to root that would increase a plant's tolerance of herbivory [24], [29], [30]. In contrast, disaccharide levels in sink tissues, systemic leaves and roots were significantly reduced (Figure 3A). The OS-elicited decrease in disaccharide levels in roots during a day may be mainly due to a reduction of sucrose because maltose levels remain low during the day and increase during the night [26], [28]. The reduced disaccharide levels in roots may have increased the gradient force driving sucrose translocation from shoot to root [24]. However, it remains an open question whether the decrease in disaccharide levels in sink leaves is linked to a reduction of sucrose or maltose levels or whether it results from increased sucrose transport from source leaves to sink leaves.

### A sugar-containing diterpene glycoside and its related genes

**Figure 4:**
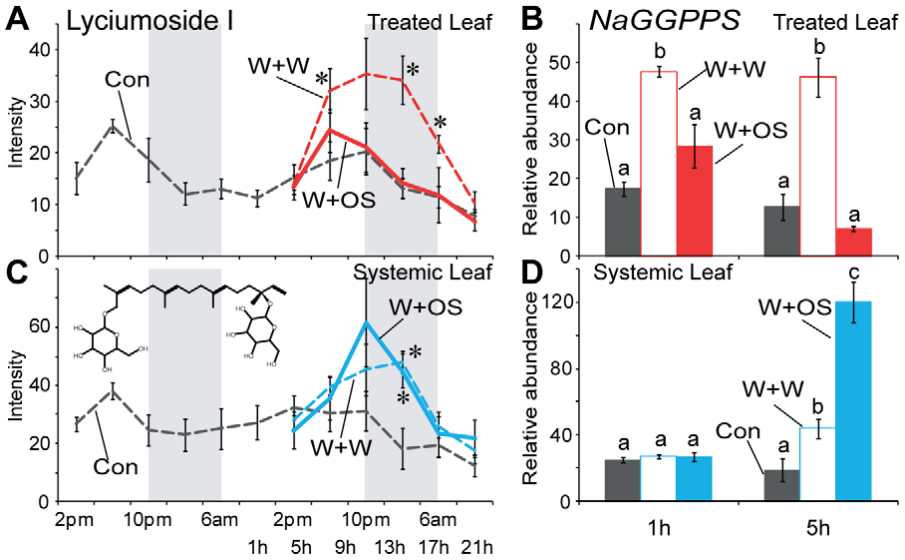
Diurnal rhythms and OS elicitation of glucose-containing secondary metabolites in different tissues. Mean (±SE) levels of normalized intensity of lyciumoside I (*m*/*z* 629.35 at 339 s, C_32_H_53_O_12_^−^) in treated (A), and untreated systemic leaves (C) at each harvest time for two days (gray dotted lines) in control plants. Lyciumoside I was not detected in roots. After W+W (dashed lines with colors) or W+OS (solid lines with colors) treatments, lyciumoside I levels were examined in treated leaves (A) and untreated systemic leaves (C). Gray boxes depict the dark period. Asterisks indicate significant differences among the treatments at the indicated time points (* = *P*<0.05, one-way ANOVA with Bonferroni *post hoc* test). (B), (D) Effects of W+W and W+OS on relative transcript abundance (±SE) of *NaGGPPS* (*N. attenuata geranylgeranyl diphosphate synthase*), a gene involved in producing the diterpenoid precursor, geranylgeranyl diphosphate [31]. Different letters (a, b and c) reflect significant differences among the treatments at the indicated time points (*P*<0.05, one-way ANOVA with Bonferroni *post hoc* test).

The analysis identified a sugar-containing defense metabolite with strong diurnal patterns of accumulation. We found that the accumulation of lyciumoside I (*m*/*z* 629.35 at 339 s, C_32_H_53_O_12_^−^), a precursor of various diterpene glycosides (DTGs) in *N. attenuata* [31], peaked at dusk in treated and systemic leaves but this metabolite was not detected in roots (Figure 4A and 4C). Sink leaves contained more lyciumoside I than did source leaves (Figure 4A and 4C). OS-elicitation did not alter the accumulation of lyciumoside I in treated leaves within 21 h, whereas W+W treatment (Figure 4A, red line) increased its levels in treated leaves at 5, 13 and 17 h (*P*<0.05, one-way ANOVA followed by Bonferroni *post hoc* test). Consistent with these results was the observation that *NaGGPPS* (*N. attenuata geranylgeranyl diphosphate synthase*) transcripts (Figure 4B), which are involved in the biosynthesis of lyciumoside I [31], significantly increased only after W+W treatment (*P*<0.05, one-way ANOVA followed by Bonferroni *post hoc* test). Systemic signaling also elicited the accumulation of lyciumoside I (Figure 4C) and *NaGGPPS* transcript (Figure 4D) in systemic leaves (*P*<0.05, one-way ANOVA followed by Bonferroni *post hoc* test). W+W and W+OS treatments resulted in similar increases in lyciumoside I, whereas OS-elicitation resulted in larger increases in *NaGGPPS* transcripts in systemic leaves after wounding (Figure 4D).

OS-elicitation reduced the wound-induced genes (type I) or amplified the regulation of genes by wounding (type II) [32]. Interestingly, *NaGGPPS* transcript showed a type I expression pattern in treated leaves and a type II expression pattern in systemic leaves. In flowering stage plants, several DTGs accumulate mainly in young leaves and reproductive tissues [31]. Induction of Lyciumoside I by OS-elicitation also followed this pattern which might enhance plant fitness by protecting these young fitness-enhancing tissues first, as predicted by Optimal Defense Theory.

### Phenylalanine/tyrosine and their related genes

**Figure 5:**
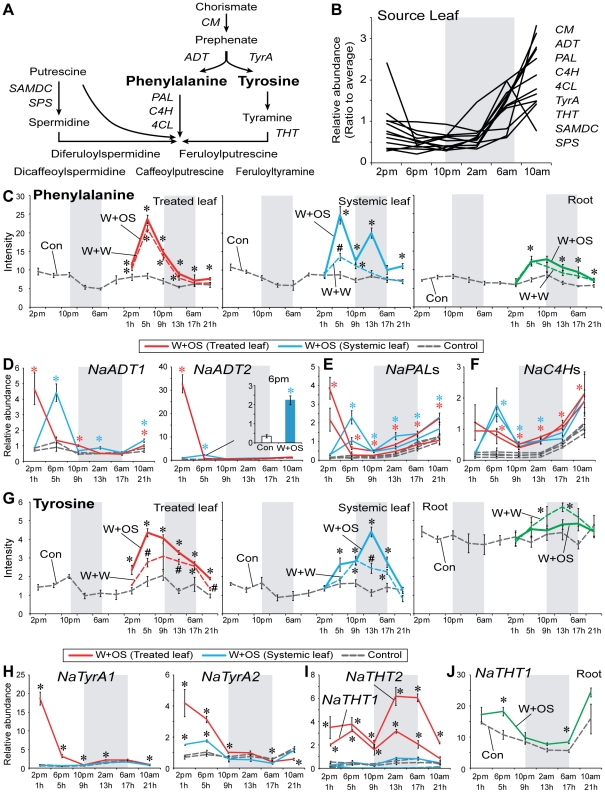
Diurnal rhythms and OS elicitation of Phe and Tyr and their related genes in different tissues. (A) Schematic overview of Phenylalanine (Phe) and Tyrosine (Tyr) metabolism. CM, chorismate mutase; ADT, arogenate dehydratase; PAL, phenylalanine ammonia lyase; C4H, cinnamate 4-hydroxylase; 4CL, 4-coumarate-coa ligase; TyrA, arogenate dehydrogenase; THT, tyramine N-hydroxycinnamoyltransferase; SAMDC, S-adenosylmethionine decarboxylase; SPDS, spemidine synthase. (B) Diurnal expression of genes encoding Phe or Tyr metabolism enzymes in source leaves. Gray box depicts the dark period. Ratio to average: Ratio of transcript abundance at the time point shown, to the mean abundance of the same transcript across all time points. Mean (±SE) levels of normalized intensity of Phe (C; *m*/*z* 164.07 at 192 s, C_9_H_10_NO_2_^−^) and Tyr (G; *m*/*z* 180.07 at 144 s, C_9_H_10_NO_3_^−^) in treated leaves, untreated leaves and roots at each harvest time for two days (gray dashed lines) in control plants. After W+W (dashed lines with colors) or W+OS (solid lines with colors) treatments, Phe (C) and Tyr (G) levels were quantified in treated leaves (red), untreated systemic leaves (blue) and roots (green). Effects of W+W and W+OS treatments on relative transcript abundance (±SE) of genes related Phe (D–F) and Tyr (H–J) metabolism. Gray box depicts the dark period. Different symbols (* and #) indicate significant differences among the treatments at the indicated time point (*P*<0.05, one-way ANOVA with Bonferroni *post hoc* test).

The aromatic amino acids, phenylalanine (Phe) and tyrosine (Tyr) are well-known primary metabolites with diurnal rhythms [9]. Phe and Tyr, which are synthesized via the shikimate pathway, are used in the production of lignin, anthocyanins, alkaloids, floral scents and defensive metabolites [33]. These compounds were also identified by our method for identifying oscillating metabolites (Figure 5C and 5G). The genes involved in their biosynthesis or catabolism showed similar diurnal patterns as well (Figure 5A, 5B and Table S3). Most of these genes increased during the day and remained at low levels at dusk and night (Figure 5B). Interestingly, Phe accumulation in different tissues peaked at different times: 2 or 6 pm in leaves and 10 pm in roots (Figure 5C and S2). While Phe levels in roots peaked later than in leaves, we did not find a diurnal pattern of the Phe biosynthetic gene transcripts, *arogenate dehydratases* (*NaADTs*) in roots, which are thought to convert arogenate to Phe, (Figure S3A and Table S3). The diurnal rhythm of Phe levels in the roots may be linked to the diurnal accumulation of *NaADT1/2* transcripts in leaves (Figure 5B and 5D) and the translocation of Phe from leaves to roots through the phloem [34]–[36]. Phe levels in flowers are particularly interesting because it is a precursor of the nocturnally emitted pollinator attractant, benzyl acetone [37], [38]. Phe levels in flowers were elevated during the night (Figure S2). Three different tissues (leaf, root and flower) have their own diurnal rhythms of Phe, which would be connected to specific roles of Phe in different tissues. Tyr levels were constitutively high in roots, exceeding the levels found in leaves even during peak accumulations (Figure 5G).

W+W and W+OS treatments induced similar increases of Phe levels in treated leaves (Figure 5C, red line; *P*<0.05, one-way ANOVA followed by Bonferroni *post hoc* test), but OS-elicitation resulted in much stronger systemic responses (Figure 5C, blue line; *P*<0.05, one-way ANOVA followed by Bonferroni *post hoc* test). Phe accumulation was also increased in roots, but the OS-elicited increase was less than in treated/systemic leaves (Figure 5C, green line). Transcripts of two *NaADT*s were significantly increased within 1 h of elicitation followed by dramatic decreases in treated leaves (Figure 5D, red line; *P*<0.05, Student's *t*-test), which may explain Phe's peak 5 h after treatment. Transcripts of the same genes (Figure 5D, blue line) were also increased in systemic leaves (*P*<0.05, Student's *t*-test) but with a 4 h delay. Phe accumulation in systemic leaves peaked twice at 5 and 13 h after OS-treatment but only once at 5 h after W+W treatment. It is possible that a part of the induced Phe accumulation in younger leaves originated from Phe produced in treated leaves.

Phenylalanine ammonia lyase (PAL) is the first enzyme in phenylpropanoid biosynthesis (Figure 5A). *NaPAL1*/*2* transcript accumulations followed a similar pattern to that of *NaADT1* (Figure 5E) with OS-elicited increases within 1 h in treated leaves and within 5 h in systemic leaves (*P*<0.05, Student's *t*-test). The other phenylpropanoid pathway gene, *C4H* (*cinnamate-4-hydrozylase*) showed a similar pattern of increase observed in *NaPAL*s transcript levels (Figure 5F; *P*<0.05, Student's *t*-test). The accumulation of *NaADT*s, *NaPAL*s and *NaC4H*s transcripts in roots was less affected by W+W and W+OS treatments than in leaves (Figure S3).

W+W and W+OS treatments increased the amount of Tyr in treated and systemic leaves (Figure 5G; *P*<0.05, one-way ANOVA followed by Bonferroni *post hoc* test), while OS-treatment increased its level with an 8 h delay in systemic compared to treated leaves (Figure 5G). Only W+W treatment increased Tyr accumulation in roots at 9 and 13 h (Figure 5G; *P*<0.05, one-way ANOVA followed by Bonferroni *post hoc* test). We identified two *arogenate dehydrogenase* (*TyrA*) genes, which are thought to be involved in Tyr biosynthesis (Figure 5H), with OS-elicited increases in treated and systemic leaves (*P*<0.05, Student's *t*-test). *NaTyrA1*/*2* transcript levels increased more in treated leaves compared to systemic leaves (Figure 5H), but induced Tyr levels in both tissues were similar (Figure 5G). The induced Tyr accumulation in treated leaves may also be translocated into systemic leaves.

Tyramine N-hydroxycinnamoyltransferase (THT) is an enzyme that conjugates cinnamoyl-CoA, caffeoyl-CoA, or feruloyl-CoA to tyramine [39]. Interestingly, two *NaTHT*s transcript levels (Figure 5I) were increased after OS-elicitation only in treated but not in systemic leaves (*P*<0.05, Student's *t*-test). *NaTHT1* (Figure 5J) and *NaTHT2* (Figure S4) transcripts accumulated to high levels in roots and only *NaTHT1* transcript displayed diurnal accumulation only in roots. OS-elicitation increased *NaTHT1* (Figure 5J) and decreased *NaTHT2* (Figure S4) transcript levels in roots (*P*<0.05, Student's *t*-test).

### Phenylpropanoid-polyamine conjugates

**Figure 6:**
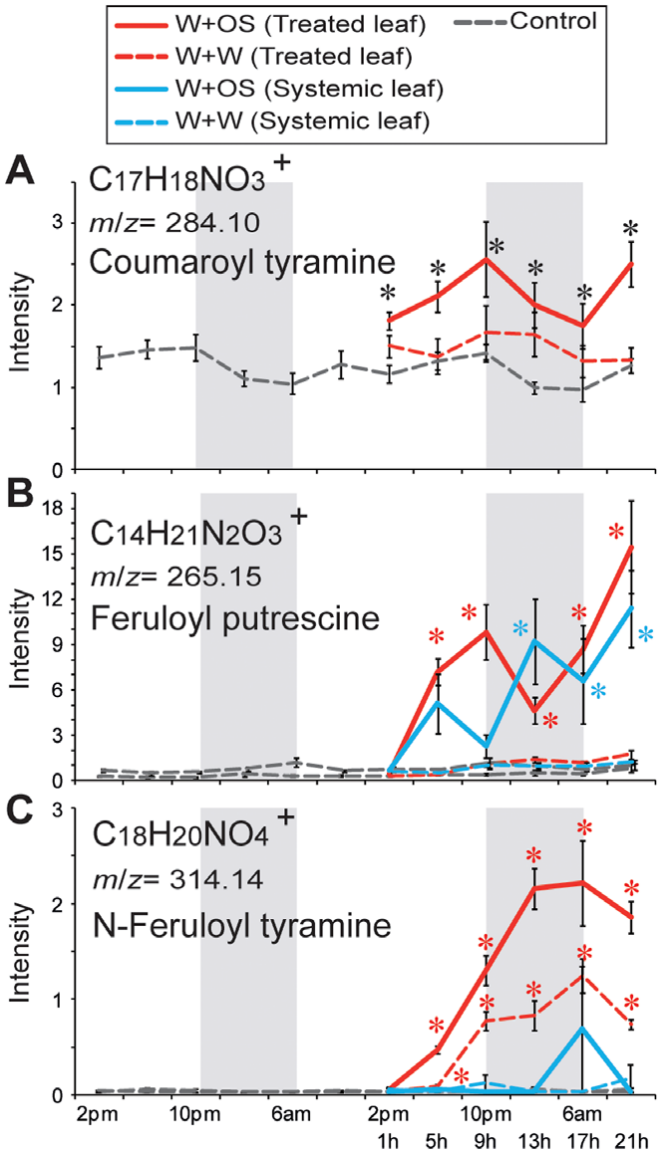
OS-elicitation affects secondary metabolites in the phenylpropanoid pathway. Mean (±SE) levels of normalized intensity of coumaroyl tyramine (A; *m*/*z* 284.10 at 165 s, C_17_H_18_NO_3_^+^), feruloyl putrescine (B; *m*/*z* 265.15 at 212 s, C_14_H_21_N_2_O_3_^+^) and N-feruloyl tyramine (C; *m*/*z* 314.14 at 319 s, C_18_H_20_NO_4_^+^) in treated and systemic leaves at each harvest time for two days (gray dotted lines) in control plants. Feruloyl putrescine and N-feruloyl tyramine were not detected in roots. After W+W (dashed lines) or W+OS (solid lines) treatments their levels were examined in treated leaves (red) and untreated systemic leaves (blue). Gray boxes depict the dark period. Asterisks indicate significant differences among the treatments at the indicated time point (* = *P*<0.05, one-way ANOVA with Bonferroni *post hoc* test).

Coumaroyl tyramine (*m*/*z* 284.10 at 165 s, C_17_H_18_NO_3_^+^) peaked during the day (Figure 6A) with an OS-elicited increase within 1 h in treated leaves (*P*<0.05, one-way ANOVA followed by Bonferroni *post hoc* test). The levels of two feruloylamine conjugates [17] were also increased by W+W or W+OS treatments (Figure 6B and 6C). The accumulation of feruloyl putrescine (*m*/*z* 265.152 at 212 s, C_14_H_21_N_2_O_3_^+^) increased in treated and systemic leaves after OS-elicitation but not after W+W treatment (Figure 6B; *P*<0.05, one-way ANOVA followed by Bonferroni *post hoc* test). N-feruloyl tyramine accumulated (*m*/*z* 314.140 at 319 s, C_18_H_20_NO_4_^+^) markedly after W+W and W+OS treatments, but only in treated leaves (Figure 6C; *P*<0.05, one-way ANOVA followed by Bonferroni *post hoc* test). Systemic leaves did not accumulate N-feruloyl tyramine after any treatments was consistent with the low accumulation of *NaTHT1*/*2* transcripts in systemic leaves (Figure 5I and 6C). However, no change in N-feruloyl tyramine levels was observed in roots (Figure S5) despite the significant increases in *NaTHT1* transcripts in these tissues (Figure 6J).

THT enzyme activity of barley and wheat is known to be stronger in roots than in shoots [40]. N-feruloyl tyramine levels in roots (Figure S5) were higher than in leaves (Figure 6C) before or after W+OS treatment, perhaps a reflection of these *NaTHT*s transcript levels, but did not show diurnal rhythms (Figure S5). Identification of oscillating tyramine conjugates in roots will be helpful to understand plant defenses against root herbivores or pathogens that have their own diurnal activity rhythms [41], [42].

### OPDA and JA in roots and their related genes

**Figure 7:**
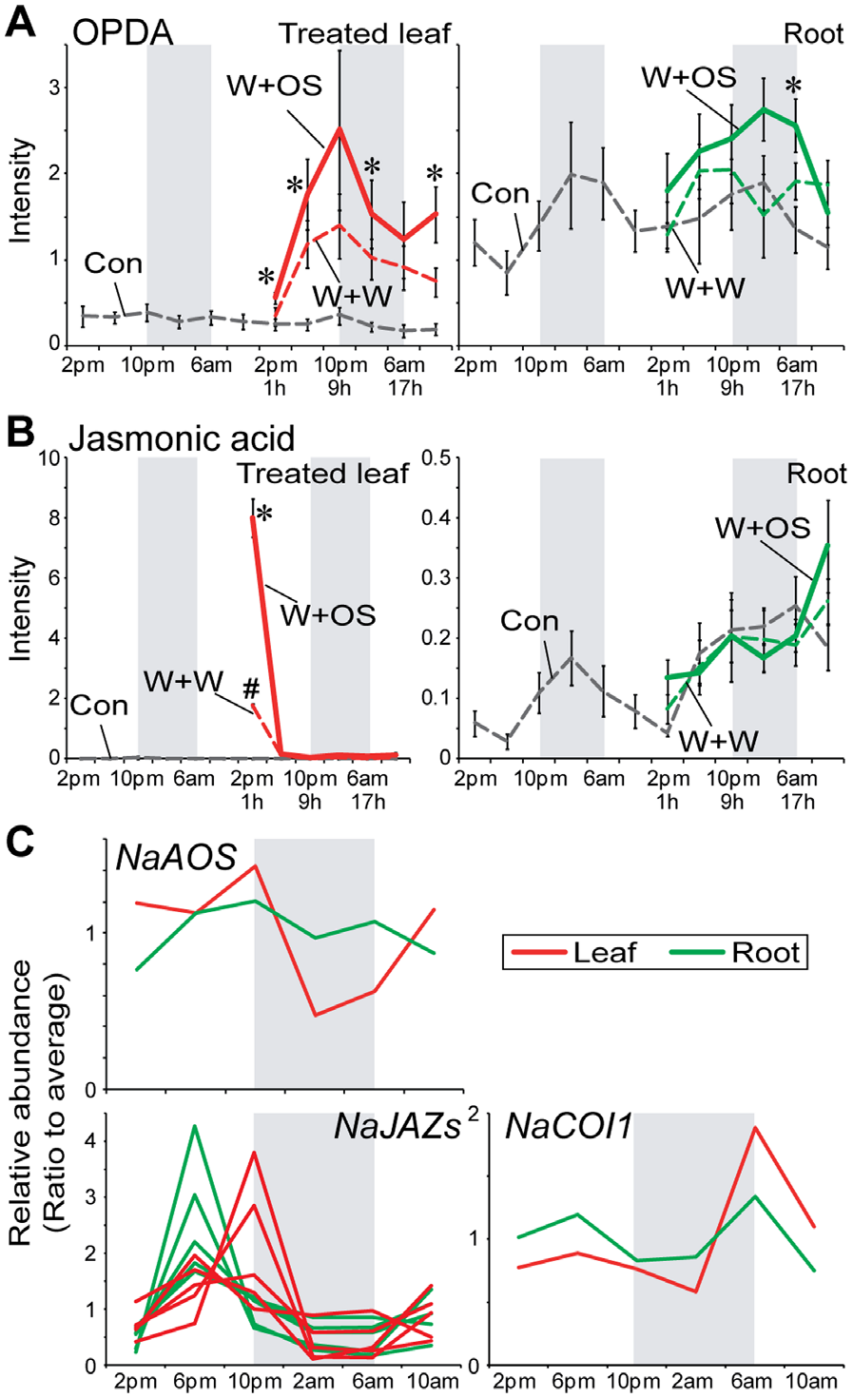
Diurnal rhythms and OS elicitation of OPDA, jasmonic acid and JA-related genes in roots. (A), (B) Mean (±SE) levels of normalized intensity of 12-oxophytodienoic acid (OPDA, *m*/*z* 291.20 at 479 s, C_18_H_27_O_3_^−^) and jasmonic acid (JA, *m*/*z* 209.17 at 368 s, C_12_H_17_O_3_^−^) in treated leaves and roots at each harvest time for two days (gray dotted lines) in control plants. After W+W (dashed lines with colors) or W+OS (solid lines with colors) treatments, their levels were examined in treated leaves (red) and roots (green). Gray boxes depict the dark period. Different symbols (* and #) indicate significant differences among the treatments at the indicated time point (*P*<0.05, one-way ANOVA with Bonferroni *post hoc* test). (C) Diurnal rhythms of gene accumulation involved in JA biosynthesis or signaling. AOS, allene oxide synthase; JAZ, jasmonate-ZIM-domain protein; COI1, coronatine insensitive 1.

We found two jasmonates with root-specific diurnal patterns of accumulation. OPDA (12-oxophytodienoic acid, *m*/*z* 291.20 at 479 s, C_18_H_27_O_3_^−^) and JA (*m*/*z* 209.12 at 368 s, C_12_H_17_O_3_^−^) levels peaked at night only in roots (Figure 7A and 7B). OPDA is a precursor of JA, which is an important phytohormone known to activate defense responses against herbivore attack [43]. JA accumulation is well known to increase after W+W and to be amplified by the OS-elicitation (Figure 7B, red line). Without OS-elicitation, OPDA and JA were detected at higher levels in roots than in leaves (Figure 7A and 7B). W+W and W+OS treatments did not dramatically alter the accumulation of OPDA or JA in roots as they did in treated leaves (Figure 7A and 7B; *P*<0.05, one-way ANOVA followed by Bonferroni *post hoc* test). Only OS-treatment slightly increased the accumulation of OPDA after 17 h (Figure 7A; *P* = 0.0064, one-way ANOVA followed by Bonferroni *post hoc* test).

We identified several oscillating transcripts involved in JA signaling (Figure 7C and Table S4).While the levels of JA in leaves did not show diurnal patterns, the JA biosynthetic genes, *AOS* (*allene oxide synthase*) and two major JA signaling components, *COI1* (*coronatine insensitive 1*) and *JAZ*s (*jasmonate-JIM-domain protein*s) transcript accumulations showed diurnal rhythms (Figure 7C, Red line). *NaCOI1* transcripts in leaves were induced at dawn and remained at low levels for the rest of the day (Figure 7C). *NaJAZ*s levels in leaves peaked at 6 or 10 pm in leaves, while the same *NaJAZ* transcripts in roots peaked at 6 pm (Figure 7C).

Fatty acid-amino acid conjugates (FACs) in *M. sexta* OS induce herbivore-specific defense responses in *N. attenuata* [16], [44]. The phytohormone, JA plays an important role in FACs-induced plant defense signaling against herbivore attack [43]. JA biosynthesis occurs in two organelles, the chloroplast and the peroxisome. α-linolenic acid released from membrane is converted into OPDA by LOX, AOS, and AOC (allene oxide cyclase), all enzymes of the chloroplast. The OPDA is then imported into the peroxisome and converted into JA [43]. Even though root cells have no chloroplasts, transcripts encoding LOX, AOS and AOC proteins are expressed in roots of several plant species, likely in the leucoplasts of roots, and jasmonates are detected in their roots [45]–[48]. To our knowledge, our study represents the first report of a diurnal rhythm of OPDA and JA in roots of plants. Basal levels of JA in leaves did not show a diurnal rhythm, although JA biosynthesis genes, *LOX3* [15] and *AOS* (Figure 7C) transcripts in *N. attenuata*'s leaves did (Figure 7C). It is still unclear whether the oscillation in JA levels in roots results from JA synthesis in leaves and transport into roots or their *de novo* synthesis in the roots. Wang *et al.* treated wounded *N. attenuata*'s leaves with isotope-labeled (^13^C_6_) Ile and detected JA-^13^C_6_-Ile in treated leaves of *N. attenuata* [49]. However, labeled JA-Ile was not detected in systemic leaves and roots. These data suggest that *N. attenuata*'s roots have the ability to *de novo* synthesize jasmonates [47], [49].

Stress-induced biosynthesis of JA initiates direct and indirect defense responses in plants [29]. The diurnal rhythm of JA levels in roots may be linked to the daily occurrence of these stresses in roots. One of the possible stresses is elicited by pathogen attack. The circadian clock component, CCA1 has been recently shown to be critical for plants in the anticipation of leaf pathogen attack and in turn regulate the expression of defense related genes against the pathogen [4]. Arabidopsis *AOS* gene has one CCA1 binding site (AAAAAATCT) and one evening element (AAAATATCT) in its promoter region [5], [50], [51]. *LOX3* in Arabidopsis have also one evening element [50], [51]. Homologous genes in *N. attenuata* showed diurnal accumulations (Figure 7C) either in leaves or in roots [15]. It will therefore be extremely interesting to analyze the promoter sequences of JA-related genes in *N. attenuata*. Leaf pathogens usually attack at dawn when temperature and moisture conditions are conducive for pathogen infection. However, roots in the soil are subjected a completely different microclimate. Diurnal rhythms of JA accumulation in roots therefore may play other roles.

Oscillating JA levels may facilitate root penetration into hard soil. The soil of *N. attenuata*'s native habitat, the Great Basin Desert in Utah is usually dry and hard. We used sand-grown roots, rather than hydroponic culture, to mimic as close as possible these natural conditions. Comparisons of the JA contents of hydroponically-grown and sand-grown roots are not simple, because these different culture conditions produce roots with different patterns of growth and morphology. Nonetheless, when we compared the JA contents in the same mass of roots, we usually observed higher JA levels in sand-grown roots than in hydroponically-grown roots (data not shown). The pattern of JA accumulation that peaked at night may be associated with the growth rhythm of roots and the production of secondary metabolites to protect root cells from infection and attack during growth-associated wounding.

Even though JA levels in leaves did not show a diurnal rhythm, JA signaling components, *COI1* and several *JAZ*s transcript levels in *N. attenuata* were diurnal-regulated (Figure 7C). Far-red light treatment increases *AOC* and *JAZ1* transcript accumulation of Arabidopsis and phytochrome A elicits the degradation of JAZ1 proteins [52], which suppress COI1-dependent JA signaling. Extrafloral nectar secretion in lima bean is regulated by JA levels but also in a light dependent manner [53]. These results suggest that diurnal changes in light quality in nature may influence JA signaling pathway in a time-dependent manner. In other words, diurnal rhythms of JA signaling components in leaves may play a central role in modulating defense responses of plants depending on the time of day when they are attacked.

### Future work

The timing of gene regulation has been intensively studied in Arabidopsis. However, a lack of association between diurnal rhythms and one of the major biotic stresses for plants, that of herbivore attack in which secondary metabolites play central roles, led us to examine how oscillating metabolites are influenced by herbivory. Recently, Kerwin *et al*. reported the reverse mechanism, that glucosinolate metabolism affects the output of the circadian clock and clock gene expression [54], [55]. Mutations in glucosinolate biosynthetic genes result in the alteration of the circadian clock genes including the *PSUDORESPONSIVE REGULATOR*s (*PRR*s). *M. sexta* attack elicits dramatic changes in *N. attenuata* metabolism [17], [21], [56]. These changes may also feedback on clock gene expression during herbivory. To test this hypothesis, in future work, we will examine the microarray data of ir*LOX2* (producing less green leaf volatiles), ir*LOX3* (deficient in JA biosynthesis), and ir*MYB8* (containing less phenylpropanoid-polyamine conjugates) of *N. attenuata* using single time point analysis described by Kerwin *et al*. [15], [54], [57].

## Materials and Methods

### Plant material and treatment

Wild type (WT), *Nicotiana attenuata* plants (30th inbred generation) were grown from seeds originating from a natural population in Utah. The original collection of the seeds was done on private lands and since *N. attenuata* is not an endangered plant species no specific permissions for seed collections were necessary. Moreover, all seeds used in this study were bred in the glasshouse at our institute. Seeds were sterilized and germinated on Gamborg's B5 medium as previously described [58]. Ten-day old seedlings were transferred to small pot (TEKU JP 3050 104 pots, Pöppelmann GmbH & Co. KG, Lohne, Germany) with Klasmann plug soil (Klasmann-Deilmann GmbH, Geesten, Germany) and after 10 days, seedlings were transferred to 1 L pots with sand to facilitate sampling of roots. Plants were watered by flood irrigation system with 200 g CaNO_3_4H_2_O, 200 g Flory B1 in 400 L water and grown in the glasshouse at 26–28°C under 16 h supplemental light from Master Sun-T PIA Agro 400 or Master Sun-T PIA Plus 600 W Na lights (Philips, Turnhout, Belgium).

We used early elongated stage of WT *N. attenuata* plants for metabolomics and transcriptomic analyses. Six biological replicates (plants) were harvested every 4 h for 2 d from each treatment group. Before freezing the samples in liquid nitrogen, roots were washed in a water tank for a few seconds to remove sand. *M. sexta* oral secretions (OS) collected from fourth- or fifth-instar larvae were diluted 1∶5 with deionized water.

### Metabolite analysis

We used a 40% methanol extraction procedure optimized for the extraction of a wide range of our interesting metabolites of in *N. attenuata* [17]. 4 µL of the resulting leaf extracts and 6 µL of the resulting root extracts were injected onto a C18 column (Acclaim, 2.2 µm particle size, 150 mm×2.1 mm inner diameter, Dionex Corporation, Sunnyvale, USA) and separated using a RSLC system (Dionex). Solvent A consisted of deionized water containing 0.1% (v/v) acetonitrile (Baker, HPLC grade) and 0.05% (v/v) formic acid. Solvent B consisted of acetonitrile and 0.05% (v/v) formic acid. The following gradient conditions were used for the chromatography: 0–0.5 min 10% B, 0.5–6.5 min linear gradient 80% B, 6.5–10 min 80% B, and re-equilibration at 10% B for 3 min. The flow rate was 300 µL/min.

An ESI-TOF mass spectrometer (Bruker Daltonic, Bremen, Germany) was used to determine the molecular mass of ionized molecular fragments and the amounts of the eluted analytes. The capillary voltage was 4500 V, and dry gas (200°C) flow rate was 8 L/min. Detected ion range was from *m*/*z* 200 to 1400 at a repetition rate of 1 Hz. The mass calibration was achieved using a sodium formate solution (10 mM sodium hydroxide and 0.2% formic acid in isopropanol/water 1∶1, v/v).

### Raw data processing

Raw data files from Bruker software (Data Analysis v4.0) were exported as netCDF format, and processed using the XCMS R package [19] (http://fiehnlab.ucdavis.edu/staff/kind/Metabolomics/Peak_Alignment/xcms/). Peak detection was performed using the centWave algorithm with the following parameter settings: ppm = 20, snthresh = 10, peakwidth = c(5,18). Retention time correction was accomplished using the XCMS retcor function with the following parameter settings: mzwid = 0.01, minfrac = 0.5, bw = 3. Missing peak data were filled using the fillPeaks function. The CAMERA package was used to annotate isotope and adduct ions (http://bioconductor.org/packages/devel/bioc/html/CAMERA.html). After 75 percentile normalization and log2 transformation, XCMS output files were processed using Microsoft Excel and the Statview software for statistical test, and TIGR's Multiexperiment Viewer software for visualization and clustering.

### Diurnal pattern analysis and metabolites annotation

The model-based HAYSTACK [20] algorithm was used to identify diurnal-regulated metabolites (http://haystack.cgrb.oregonstate.edu/). Isotope ions detected using CAMERA were removed before processing using pattern matching algorithm of HAYSTACK. With the models from HAYSTACK we selected oscillating metabolites, typically with the following values in the selection filters: correlation cutoff 0.8, fold cutoff 1.5, *P*-value cutoff 0.05. Molecular formulas were generated using the SmartFormular algorithm in Data Analysis v4.0 software (Bruker). The following maximum elemental composition C_a_H_b_N_c_O_d_Na_e_K_f_ and restrictions were used: 1≤b/a≤3; e = 0 or 1; f = 0 or 1; a, b, c and d not limited. Rings plus double bonds values from −0.5 to 40, the nitrogen rule and ions of even electron configuration were considered. The structural annotation based on tandem MS measurements of the precursor ions of selected diterpene glucosides and feruloyl putrescine has been published by our group in Gaquerel *et al*. [17]. Tandem MS measurements for these metabolites are available in the supplemental online material associated with this article. Lyciumoside I has initially been characterized in *N. attenuata* by means of NMR in Heiling *et al*. [31]. Phenylalanine, tyrosine, 12-oxo-phytodienoic acid and jasmonic acid were identified after comparison with authentic standard material. Manual annotation using metabolite information from the literature was performed in the case of tyramine conjugates. Feruloyl tyramine and coumaroyl tyramine are well described in tobacco cells' metabolites whose biosynthesis highly increases by wounding [59], [60]. Besides elemental formula calculation, the annotation of these metabolites was facilitated by typical ion signatures corresponding to coumaroyl- (*m/z* 147.04) and feruloyl- (*m/z* 177.04) residues released after the break of the ester bound linking them to a core tyramine molecule during in-source fragmentation.

### Microarray data and analysis

Three biological replicates among the six replicates harvested at each harvest time used for metabolites analysis were also used for RNA isolation (six harvest time for control and W+OS treatments, three harvest time for W+W treatments). Total RNA was isolated with TRIZOL reagent and labeled cRNA with the Quick Amp labeling kit (Agilent). Each sample was hybridized on Agilent single color technology arrays (4×44K 60-mer oligonucleotide microarray designed for *N. attenuata* transcriptome analysis, http://www.agilent.com, GEO accession number GPL13527). Agilent microarray scanner (G2565BA) and Scan Control software were used to obtain intensity of the spots. All microarray data with each probe name were deposited in the NCBI GEO database (accession number GSE30287). We confirm that all details are MIAME compliant. The resulting gene expression profiles were analyzed using GeneSpring GX software (Silicon Genetics, Redwood City, CA). Raw intensities were normalized using the 75th percentile value and log2 and baseline transformed prior statistical analysis. Probes were filtered based on their Quality control Metrics.

The HAYSTACK algorism was also used to examine the diurnal rhythm of genes after we selected genes of interest involved in biosynthesis or signaling of oscillating metabolites. With the models from HAYSTACK we examine the diurnal rhythm of gene of interest, typically with the following values in the selection filters: correlation cutoff 0.8, fold cutoff 2, *P*-value cutoff 0.05.

## Supporting Information

Figure S1 **Accumulation of** ***m*** **/** ***z*** **= 683.23 at retention time, 90 s, in roots.** Mean (±SE) levels of normalized intensity of *m*/*z* = 683.23 at 90 s in roots. Calculated molecular formula (C_24_H_43_O_22_^−^) and retention time (90 s) indicated that it is a disaccharide dimer. After W+W (dashed lines with colors) or W+OS (solid lines with colors) treatments, compound levels were examined in roots (green). Gray boxes depict the dark period. Asterisks indicate significant differences among the treatments at the indicated time point (* = *P*<0.05, one-way ANOVA with Bonferroni *post hoc* test).
(TIF)

Figure S2 **Accumulation of Phe in leaves, roots and open flowers.** Mean (±SE) levels of normalized intensity of Phe in different tissues. Phe accumulation was quantified in leaves (red), roots (green), and flowers (black). To calculate relative accumulation, 75th percentile normalized intensity at each harvest time was divided by average value over all time points. We collected open flowers from 7 week-old plants and extracted metabolites with a 40% methanol extraction method. Gray box depicts the dark period.
(TIF)

Figure S3 **Transcript abundance of** ***NaADT1*** **/** ***2*** **,** ***NaPAL1*** **/** ***2*** **, and** ***NaC4H1*** **/** ***2*** **in roots.** Mean (±SE) levels of normalized intensity of *NaADT*s, *NaPAL*s and *NaC4H*s in roots. ADT, arogenate dehydratase; PAL, phenylalanine ammonia lyase; C4H, cinnamate 4-hydroxylase. Gray boxes depict the dark period. Asterisks indicate significant differences between control plants and *M. sexta* oral secretions-treated plants (W+OS) at indicated time points (* = *P*<0.05, as determined by Student's *t*-test).
(TIF)

Figure S4 **Transcript abundance of** ***NaTHT2*** **in roots.** Mean (±SE) levels of normalized intensity of *NaTHT2* in roots. THT, tyramine N-hydroxycinnamoyltransferase. Gray box depicts the dark period. Asterisks indicate significant differences between control plants and *M. sexta* oral secretions-treated plants (W+OS) at indicated time points (* = *P*<0.05, as determined by Student's *t*-test).
(TIF)

Figure S5 **Accumulation of N-feruloyl tyramine in roots.** Mean (±SE) levels of normalized intensity of N-feruloyl tyramine in roots. Gray boxes depict the dark period. Asterisks indicate significant differences between control plants and *M. sexta* oral secretions-treated plants (W+OS) at indicated time points (* = *P*<0.05, as determined by Student's *t*-test).
(TIF)

Table S1 **Proposed molecular formulas for oscillating compounds detected in leaves and roots.**
(TIF)

Table S2 **Oscillating transcripts involved in sugar metabolism of** ***N. attenuata*** **.**
(TIF)

Table S3 **Oscillating transcripts involved in Phe/Tyr metabolism of** ***N. attenuata*** **.**
(TIF)

Table S4 **Oscillating transcripts involved in jasmonic acid metabolism and signaling of** ***N. attenuata*** **.**
(TIF)

File S1 **Complete list and expression value of oscillating metabolites and its related transcripts.**
(XLSX)
