# Supplementary figures and images for: Tissue Specific Diurnal Rhythms of Metabolites and Their Regulation during Herbivore Attack in a Native Tobacco, Nicotiana attenuata
Source: PLoS One. 2011 Oct 18;6(10):e26214. doi: 10.1371/journal.pone.0026214 (PMC3196511; doi:10.1371/journal.pone.0026214)

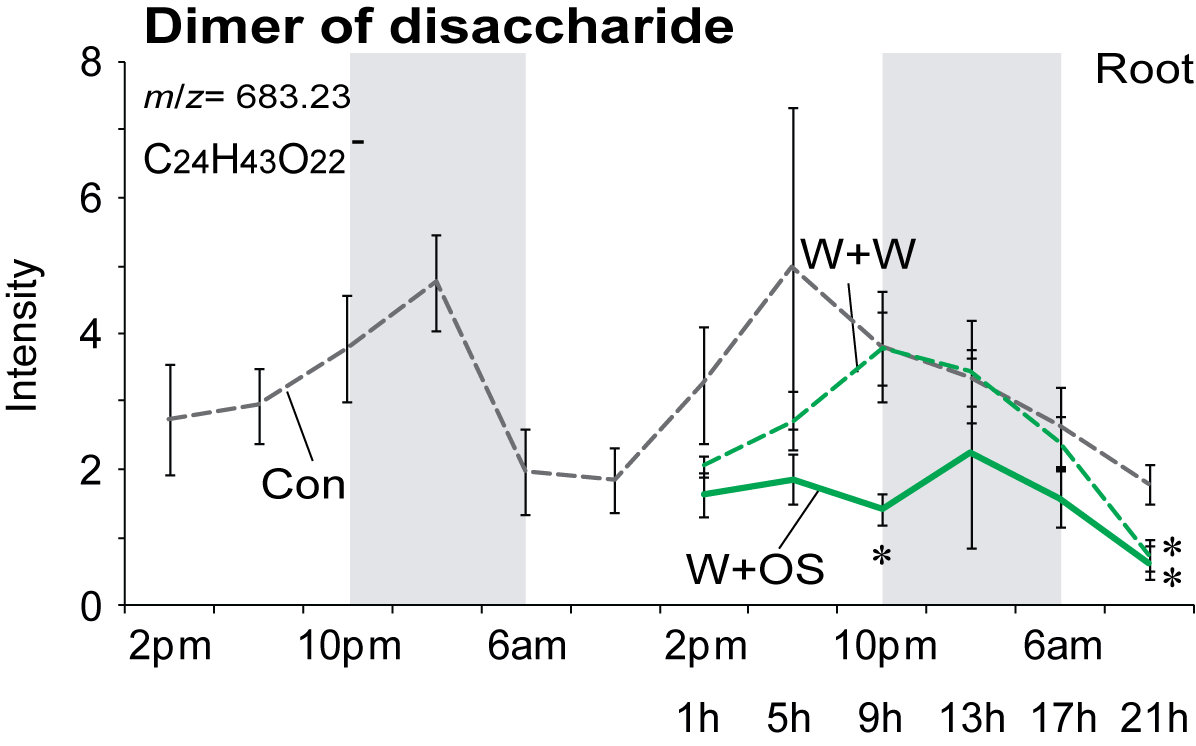

Supplement: Figure S1 — Accumulation of m / z = 683.23 at retention time, 90 s, in roots. Mean (±SE) levels of normalized intensity of m/z = 683.23 at 90 s in roots. Calculated molecular formula (C24H43O22 −) and retention time (90 s) indicated that it is a disaccharide dimer. After W+W (dashed lines with colors) or W+OS (solid lines with colors) treatments, compound levels were examined in roots (green). Gray boxes depict the dark period. Asterisks indicate significant differences among the treatments at the indicated time point (* = P<0.05, one-way ANOVA with Bonferroni post hoc test). (TIF) [file pone.0026214.s001.tif]

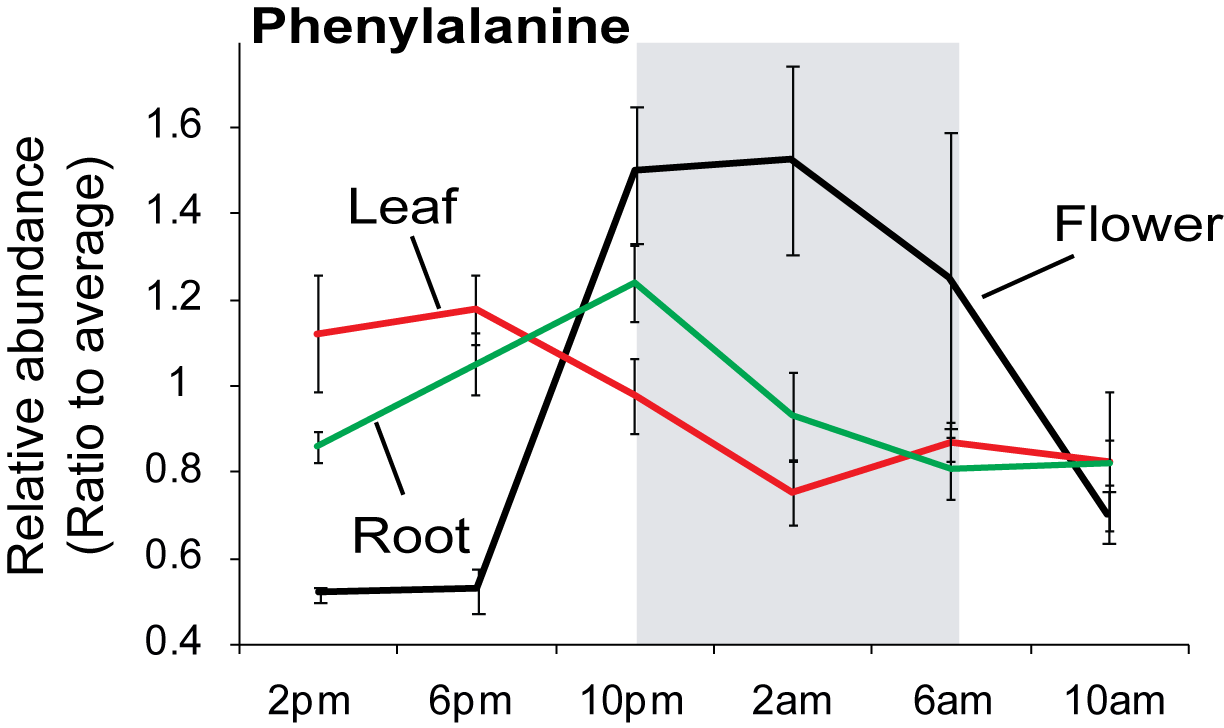

Supplement: Figure S2 — Accumulation of Phe in leaves, roots and open flowers. Mean (±SE) levels of normalized intensity of Phe in different tissues. Phe accumulation was quantified in leaves (red), roots (green), and flowers (black). To calculate relative accumulation, 75th percentile normalized intensity at each harvest time was divided by average value over all time points. We collected open flowers from 7 week-old plants and extracted metabolites with a 40% methanol extraction method. Gray box depicts the dark period. (TIF) [file pone.0026214.s002.tif]

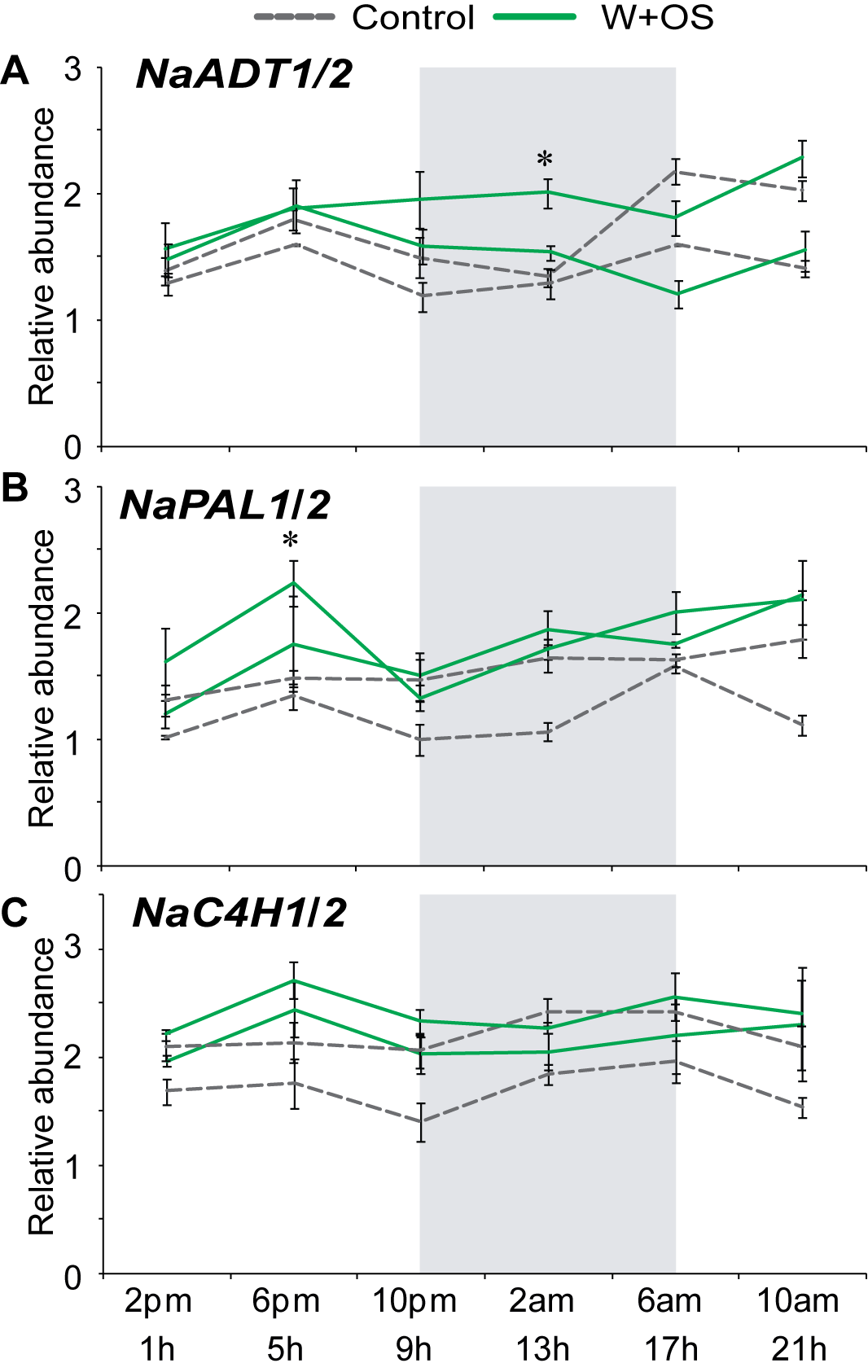

Supplement: Figure S3 — Transcript abundance of NaADT1 / 2 , NaPAL1 / 2 , and NaC4H1 / 2 in roots. Mean (±SE) levels of normalized intensity of NaADTs, NaPALs and NaC4Hs in roots. ADT, arogenate dehydratase; PAL, phenylalanine ammonia lyase; C4H, cinnamate 4-hydroxylase. Gray boxes depict the dark period. Asterisks indicate significant differences between control plants and M. sexta oral secretions-treated plants (W+OS) at indicated time points (* = P<0.05, as determined by Student's t-test). (TIF) [file pone.0026214.s003.tif]

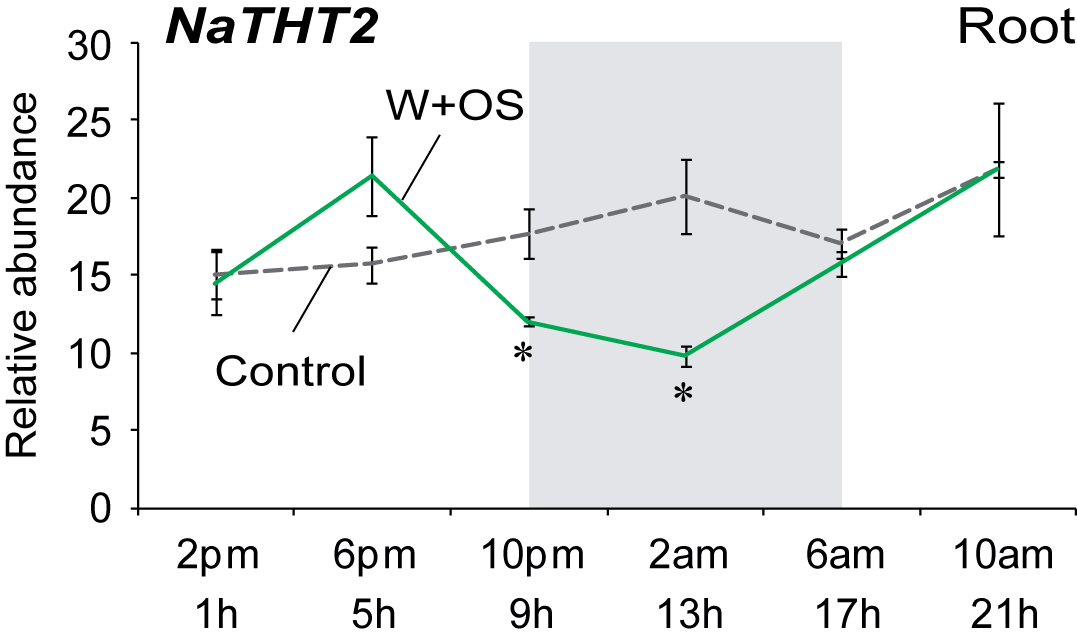

Supplement: Figure S4 — Transcript abundance of NaTHT2 in roots. Mean (±SE) levels of normalized intensity of NaTHT2 in roots. THT, tyramine N-hydroxycinnamoyltransferase. Gray box depicts the dark period. Asterisks indicate significant differences between control plants and M. sexta oral secretions-treated plants (W+OS) at indicated time points (* = P<0.05, as determined by Student's t-test). (TIF) [file pone.0026214.s004.tif]

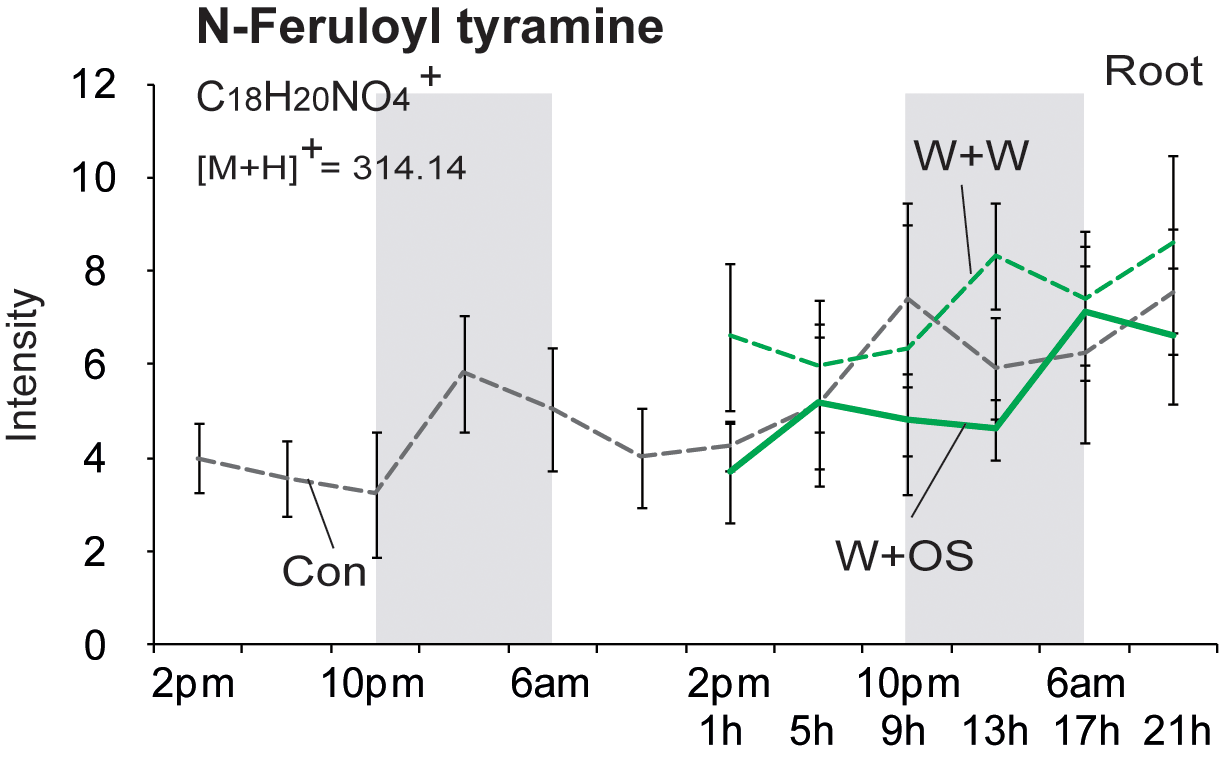

Supplement: Figure S5 — Accumulation of N-feruloyl tyramine in roots. Mean (±SE) levels of normalized intensity of N-feruloyl tyramine in roots. Gray boxes depict the dark period. Asterisks indicate significant differences between control plants and M. sexta oral secretions-treated plants (W+OS) at indicated time points (* = P<0.05, as determined by Student's t-test). (TIF) [file pone.0026214.s005.tif]

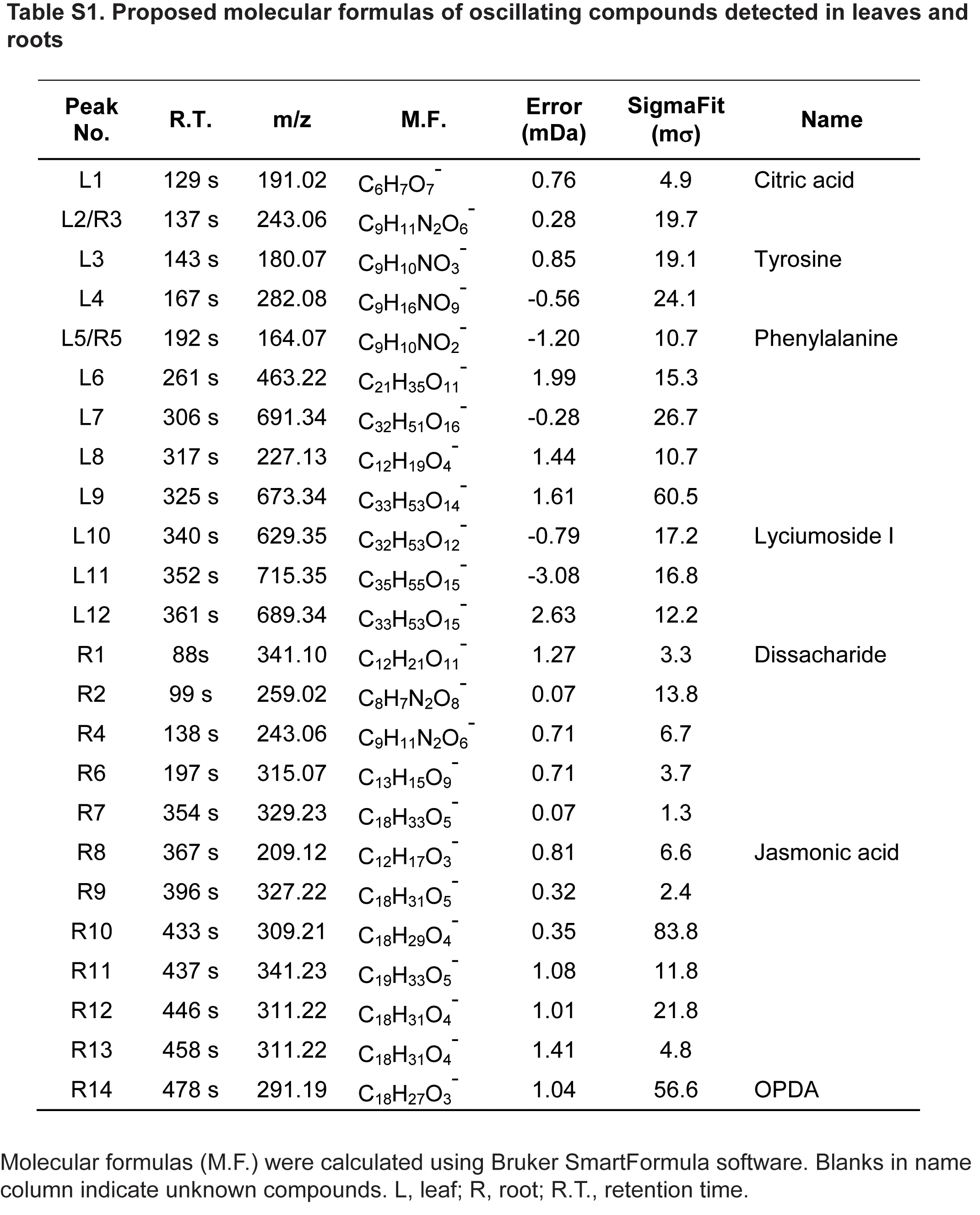

Supplement: Table S1 — Proposed molecular formulas for oscillating compounds detected in leaves and roots. (TIF) [file pone.0026214.s006.tif]

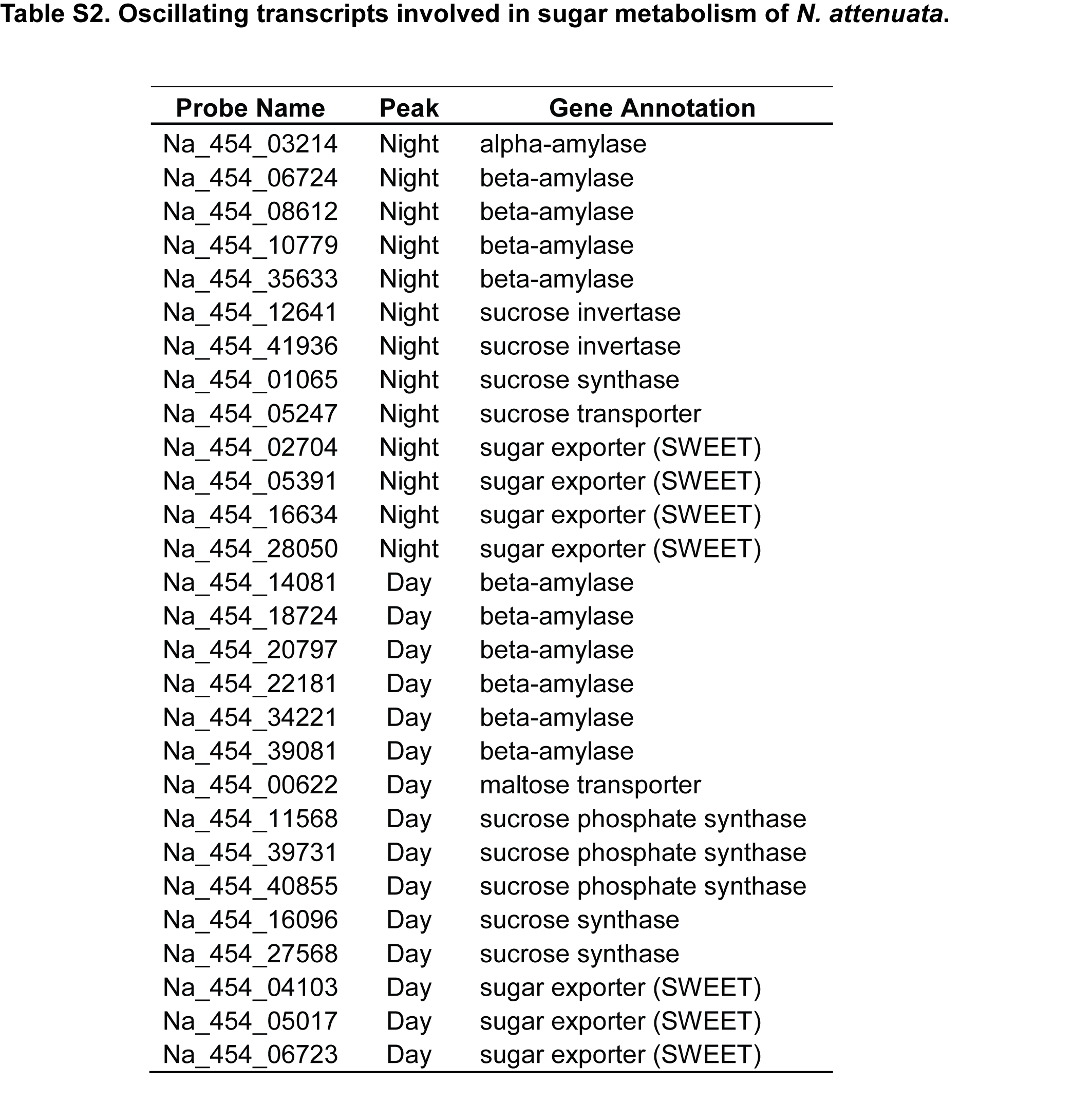

Supplement: Table S2 — Oscillating transcripts involved in sugar metabolism of N. attenuata . (TIF) [file pone.0026214.s007.tif]

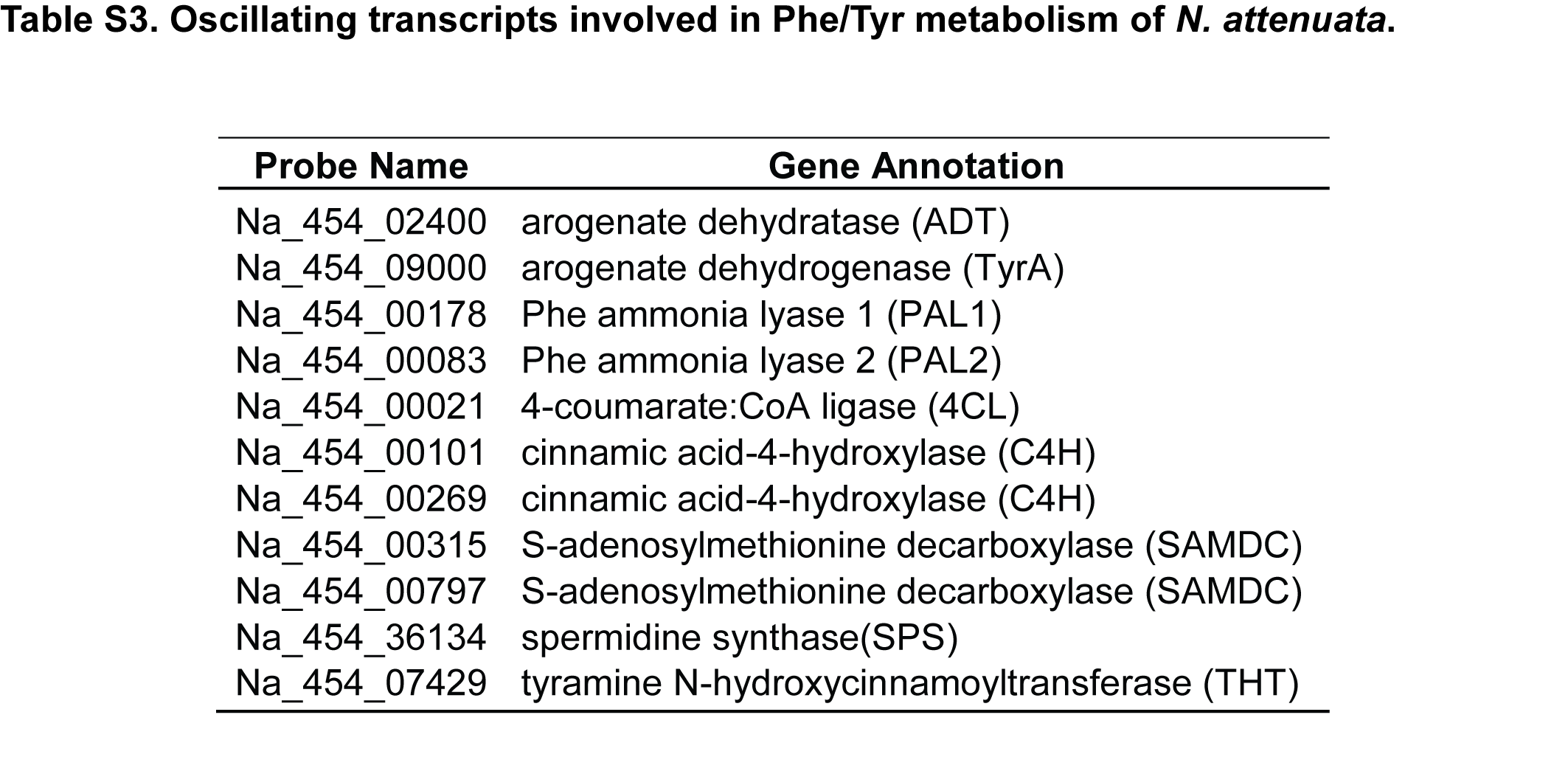

Supplement: Table S3 — Oscillating transcripts involved in Phe/Tyr metabolism of N. attenuata . (TIF) [file pone.0026214.s008.tif]

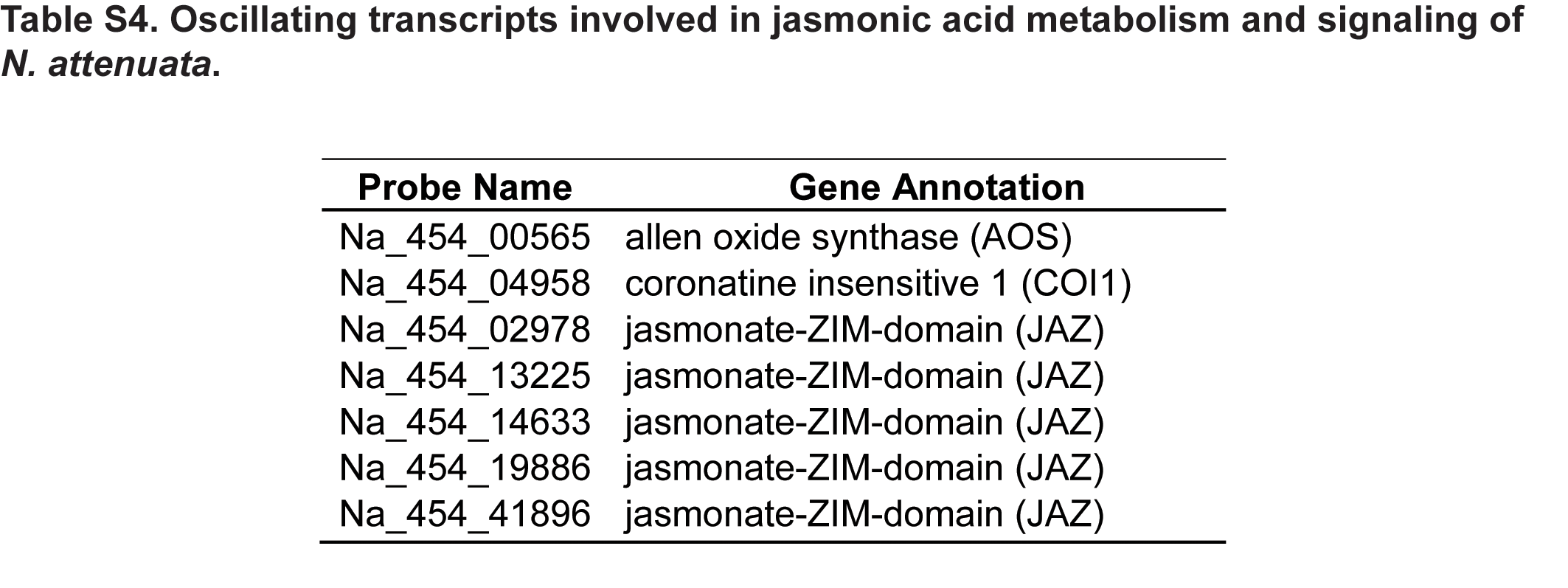

Supplement: Table S4 — Oscillating transcripts involved in jasmonic acid metabolism and signaling of N. attenuata . (TIF) [file pone.0026214.s009.tif]
